# Psychological determinants of pro-environmental behavior among environmental volunteers in Taiwan: a TPB and VBN approach

**DOI:** 10.3389/fpsyg.2025.1670383

**Published:** 2026-01-21

**Authors:** Su Hwa Lin, Amit Kumar Sah, Yao-Ming Hong

**Affiliations:** 1 Department of Science Education and Application, National Taichung University of Education, Taichung, Taiwan; 2 Department of Natural Resources and Environmental Studies, National Dong Hwa University, Hualien, Taiwan

**Keywords:** action skills, behavioral predictors, environmental literacy, environmental psychology, pro-environmental behavior, TPB, VBN, volunteer engagement

## Abstract

**Introduction:**

Understanding the psychological mechanisms that drive pro-environmental behavior is essential for advancing environmental psychology and improving volunteer-based environmental programs. This study investigates how cognitive, affective, and behavioral components of environmental literacy shape ecological action among environmental education volunteers in Taiwan, with the objective of identifying sequential pathways linking environmental awareness, knowledge, attitudes, action skills, and pro-environmental behavior, as well as examining whether demographic characteristics influence these pathways.

**Methods:**

Guided by the Theory of Planned Behavior (TPB) and Value-Belief-Norm (VBN) models, a structured questionnaire was administered to 921 certified environmental education volunteers across Chiayi, Taichung, and Hualien. The sample comprised 62.4% females and 37.6% males, with a mean age of 53.7 years (SD = 12.8). Measures included environmental awareness, knowledge, attitudes, action skills, self-reported behavior, and demographic variables. Hierarchical regression and moderation analyses were conducted.

**Results:**

Hierarchical regression analyses revealed a robust sequential pattern in which environmental awareness significantly predicted knowledge (β = 0.41, *p* < 0.001), knowledge shaped attitudes (β = 0.36, *p* < 0.001), attitudes enhanced action skills (β = 0.39, *p* < 0.001), and action skills emerged as the strongest predictor of pro-environmental behavior (β = 0.47, *p* < 0.001). Moderation analyses further indicated that age, gender, and education level did not significantly influence these pathways (all *p*>0.05), demonstrating a consistent psychological structure across demographic groups.

**Discussion:**

These findings validate TPB and VBN mechanisms within community-based environmental education and highlight the critical role of procedural competence and perceived behavioral control in transforming intention into action. Practically, the results provide evidence-based direction for governmental agencies, NGOs, and community organizations to design training programs that integrate skill-building, strengthen volunteer self-efficacy, and enhance the effectiveness of environmental action in real-world sustainability initiatives.

## 1 Introduction

Environmental issues such as climate change, biodiversity loss, waste mismanagement, and air and water pollution have intensified globally, necessitating urgent, large-scale behavioral transformation (Farida, 2019; McBride et al., 2013). In this context, environmental literacy—defined as a multidimensional construct encompassing awareness, knowledge, attitudes, action skills, and behavior—has emerged as a critical psychological framework for promoting sustainability-oriented decision-making and civic engagement (Hollweg et al., 2011). Environmental literacy is not merely an educational outcome; it represents a sequence of cognitive, affective, and behavioral competencies that collectively shape pro-environmental behavior.

In Taiwan, the enactment of the Environmental Education Act (2010) institutionalized environmental education across schools, governmental bodies, and community settings, reinforcing public engagement through structured learning opportunities (Ho et al., 2017; Ye and Shih, 2020). Among these initiatives, certified environmental education volunteers have played a central yet under-researched role. These individuals undergo formal training and serve as community intermediaries who translate abstract policy goals into local action by organizing events, advocating for sustainability, and mobilizing peers (Ding and Schuett, 2020). Despite their critical role in grassroots environmental efforts, little empirical research has examined the psychological profiles and behavioral pathways of these volunteers. Environmental education volunteers in Taiwan represent a distinct population whose behavioral dynamics differ from those of the general public. Unlike ordinary citizens, volunteers complete a formal certification process that includes structured training, competency assessments, practical fieldwork, and continuing education requirements mandated under the Environmental Education Act. Their responsibilities extend beyond passive environmental concern; they are entrusted with tasks such as conducting ecological monitoring, guiding environmental interpretation activities, assisting public agencies during environmental inspections, and coordinating community mobilization efforts. These duties require both procedural competence and an internalized sense of responsibility, making volunteers uniquely positioned to translate environmental policy into concrete community action. Understanding their psychological mechanisms is therefore essential not only for academic purposes but also for strengthening Taiwan's community-based environmental governance system. Prior studies have focused predominantly on students (Tikka et al., 2000), teachers (Liu et al., 2015), or the general public (Lin et al., 2025; Zsóka et al., 2013), leaving a gap in understanding how volunteers internalize and act upon environmental concerns.

To address this gap, the present study investigates environmental literacy among 921 certified volunteers in three socio-geographically distinct regions of Taiwan—Chiayi (rural), Taichung (urban), and Hualien (semi-urban). Adopting a psychological lens, the study analyzes how five core components—environmental awareness, knowledge, attitudes, action skills, and self-reported behavior—interrelate within a behavioral framework. Drawing on the theory of planned behavior (Ajzen, 1991) and the value-belief-norm (VBN) theory (Stern et al., 1999), the study models how internal cognitive and normative processes shape individual-level environmental engagement.

The theory of planned behavior (TPB) provides a strong foundation for understanding how volunteers translate environmental intention into actual behavior. TPB posits that attitudes toward a behavior, subjective norms, and perceived behavioral control jointly shape behavioral intentions, which subsequently guide action (Ajzen, 1991). In volunteer-based environmental work, perceived behavioral control is especially salient because volunteers frequently engage in tasks that require procedural competence, such as documenting ecological changes, conducting environmental patrols, or leading community education programs. Research has shown that individuals are more likely to perform pro-environmental behaviors when they believe they have the necessary skills and the ability to overcome practical barriers (Kaiser and Fuhrer, 2003). Within the context of environmental literacy, action skills represent a direct behavioral expression of perceived behavioral control, meaning that TPB helps explain why volunteers with stronger procedural skills are more likely to engage in sustained ecological action. Thus, TPB is particularly relevant in this study because it captures the capability-based and confidence-driven components that underlie volunteers' behavioral performance.

The value-belief-norm (VBN) theory complements TPB by addressing the motivational and moral foundations of environmental action. VBN argues that individuals' pro-environmental behavior arises from a chain of influences beginning with fundamental values, which shape ecological worldviews, activate awareness of environmental consequences, and strengthen personal norms that motivate moral obligation to act (Stern, 2000; Stern et al., 1999). Environmental volunteers often enter service with strong biospheric or altruistic values and a heightened sense of responsibility for ecological stewardship, making VBN particularly relevant to their behavioral engagement. Studies have demonstrated that individuals with strong environmental values and activated personal norms are more likely to volunteer, advocate for sustainability, and persist in environmental actions even when external incentives are absent (De Groot and Steg, 2008; Thøgersen and Ölander, 2006). In the present study, components such as environmental awareness and attitudes reflect VBN's emphasis on value-driven and norm-based motivation. By integrating VBN, the study captures the internal moral drivers that shape volunteers' commitment to environmental protection, complementing the ability-based mechanisms emphasized by TPB.

More specifically, the TPB emphasizes the role of intention, attitude, and perceived behavioral control in shaping behavior, while the VBN model highlights how value activation and personal norms motivate moral environmental action. While both theories have been widely applied independently, few studies have connected their cognitive, normative, and skill-based components within a single framework or examined how these mechanisms operate in volunteer-based environmental governance systems. Our contribution lies in adapting and combining these established theories to address an underexplored population and behavioral context, rather than introducing methodological or statistical innovations. Recent meta-analyses have further reinforced these models by demonstrating that environmental concern functions as a significant moderator and enhancer of behavioral intention—amplifying the effects of TPB constructs across diverse populations (Lucarelli et al., 2020). This dual-theory approach thus allows for a comprehensive exploration of both rational decision-making and affective motivational factors in environmental volunteerism. In addition, this study evaluates the moderating influence of demographic variables—age, gender, and education—which are known to affect environmental perceptions and motivations (Chawla, 1999; Wray-Lake et al., 2010; Zelezny et al., 2000).

Recent work has also begun to integrate motivational constructs with TPB to better account for environmental intentions. Cai et al. (2025b) combined Self-Determination Theory with the theory of planned behavior to examine how self-determined efficacy influences university students' environmental conservation intentions, using a hybrid PLS-SEM and artificial neural network (ANN) approach. Their findings indicate that autonomy, competence, and interpersonal relationships operate through attitudes, subjective norms, and perceived behavioral control to strengthen conservation intentions, highlighting both rational-cognitive and motivational pathways in pro-environmental behavior. Building on this line of research, the present study extends the focus from university students to certified environmental volunteers and examines how environmental literacy components (awareness, knowledge, attitudes, and action skills) align with TPB- and VBN-based mechanisms in a community-based volunteer context.

Conceptually, this research is anchored in an integrative framework of environmental literacy. Awareness functions as the perceptual and affective base for recognizing environmental change (Abd-Rahman et al., 2022; Hungerford and Volk, 1990), while knowledge provides the cognitive structure for understanding environmental systems, actions, and outcomes (Frick et al., 2004; Kaiser and Fuhrer, 2003). Attitudes reflect emotional and evaluative predispositions toward sustainability (Bamberg and Möser, 2007; Milfont, 2012), and action skills represent the procedural competencies required to operationalize intention (Jensen and Schnack, 2006; Kolb, 1984; Steg and Vlek, 2009). Behavior, as the final outcome, manifests through both private and collective environmental practices (Otto and Pensini, 2017; Stern et al., 1999).

Recent empirical studies also highlight the need for multi-layered approaches to understanding environmental behavior. For example, Di et al. (2024) and Sun et al. (2025) demonstrated that environmental engagement and low-carbon behavior are shaped by intertwined cognitive, normative, and contextual factors, emphasizing the value of integrated theoretical perspectives. Although these studies used advanced analytical techniques such as SEM–ANN, their findings support the broader argument that complex environmental behaviors require frameworks that account for both rational decision-making and value-driven motivations—an approach consistent with the combined use of TPB and VBN in the present study.

By mapping these components and testing their interrelations across diverse demographic groups, this study offers both theoretical and applied insights. Demographic characteristics such as age, gender, and education can shape how individuals perceive environmental issues and translate psychological factors into action. Prior research shows that people of different ages, educational backgrounds, and genders may vary in their environmental knowledge, concern, and readiness to act (Wray-Lake et al., 2010; Zelezny et al., 2000). Since Taiwan's certified volunteers come from diverse demographic groups, examining these variables as moderators helps determine whether the psychological pathways identified in this study operate similarly across subpopulations or differ based on individual characteristics. It advances environmental psychology by empirically validating sequential behavioral models among an organized volunteer population and informs the development of targeted interventions that enhance perceived behavioral control, personal norms, and skill-building. In doing so, the study contributes not only to Taiwan's environmental literacy initiatives but also to broader international goals of cultivating psychologically informed pathways to sustainable behavior.

## 2 Hypotheses and research methodology

### 2.1 Direct hypotheses

Understanding how the dimensions of environmental literacy—awareness, knowledge, attitudes, action skills, and behavior—interact is essential for evaluating volunteer-based environmental education programs. Building on robust theoretical frameworks such as the theory of planned behavior (Ajzen, 1991), the value-belief-norm theory (Stern et al., 1999), and the environmental behavior model (Hines et al., 1987), this study formulates a comprehensive set of direct hypotheses. These are further informed by empirical studies from both global and Taiwanese contexts, including insights from Taiwan's Environmental Education Act (Ye and Shih, 2020).

**H1:** Environmental awareness (EA) positively influences environmental knowledge (EK). Awareness stimulates interest in factual understanding and drives individuals to seek more information about ecological problems (Hollweg et al., 2011; Hungerford and Volk, 1990; McBride et al., 2013).

**H2:** Environmental knowledge (EK) positively influences environmental attitudes (EAT). Knowledge provides the cognitive foundation for evaluative and normative stances, deepening concern and shaping pro-environmental attitudes (Ajzen, 1991; Kollmuss and Agyeman, 2002; Singleton, 2017).

**H3:** Environmental knowledge (EK) positively influences environmental action skills (EAS). Understanding procedures and environmental systems equips individuals to undertake specific actions, strengthening their practical capacities (Frick et al., 2004; Kaiser and Fuhrer, 2003; Steg and Vlek, 2009).

**H4:** Environmental attitudes (EAT) positively influence environmental action skills (EAS). Concern and moral obligation motivate individuals to acquire and practice skills aligned with their values (Hsu et al., 2019; Kaiser and Fuhrer, 2003; Kals et al., 1999).

**H5:** Environmental action skills (EAS) positively influence environmental behavior (EB). Action skills translate psychological readiness into observable engagement, bridging intention and consistent environmental practice (Hines et al., 1987; Jensen and Schnack, 2006; Stern, 2000).

**H6:** Environmental attitudes (EAT) positively influence environmental behavior (EB). Values and beliefs directly sustain environmental behaviors, emphasizing the attitudinal basis of consistent practices (Bamberg and Möser, 2007; Gifford and Nilsson, 2014).

### 2.2 Moderating hypotheses

Although these direct relationships are theoretically robust, demographic factors likely moderate these pathways.

**H7:** Age moderates the relationship between EA and EK. Older individuals may draw on accumulated experiences, while younger individuals engage more through formal education and digital platforms, influencing how awareness leads to knowledge (Zsóka et al., 2013).

**H8:** Gender moderates the relationship between EAT and EAS. Women often report higher environmental concern yet face societal barriers to translating attitudes into practical skills, whereas men may display greater confidence in undertaking environmental tasks despite slightly weaker attitudes (Zelezny et al., 2000).

**H9:** Education level moderates the relationship between EK and EAT. Higher education reinforces the integration of knowledge into personal value systems, strengthening environmental attitudes (Tikka et al., 2000).

These moderating effects highlight the importance of tailoring environmental education programs to the diverse backgrounds of volunteers. Taiwan's volunteer culture, which integrates local identity and stewardship, may further amplify these relationships by embedding environmental learning within cultural and community contexts.

**Figure 1:**
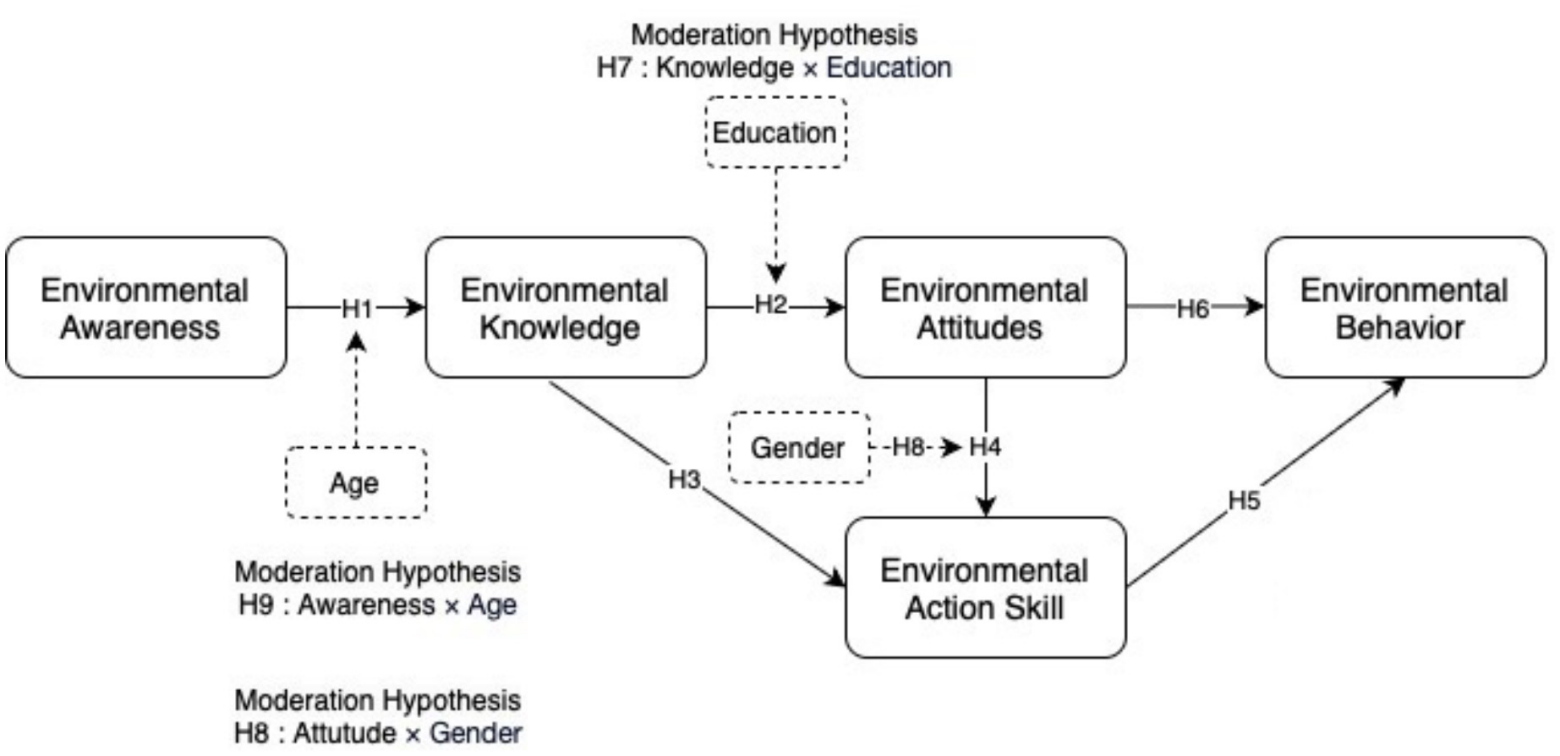
Conceptual framework illustrating hypothesized direct and moderated relationships, grounded in TPB, VBN, and the environmental behavior model.

The conceptual framework in Figure 1 summarizes these hypothesized relationships. It illustrates how environmental awareness, knowledge, attitudes, action skills, and behavior are interconnected, while also showing the moderating roles of age, gender, and education level. This structure establishes a rigorous empirical and theoretical foundation for the analysis of how volunteer environmental literacy develops and varies across demographic groups, providing essential guidance for the study's methodological and analytical design.

**Figure 2:**
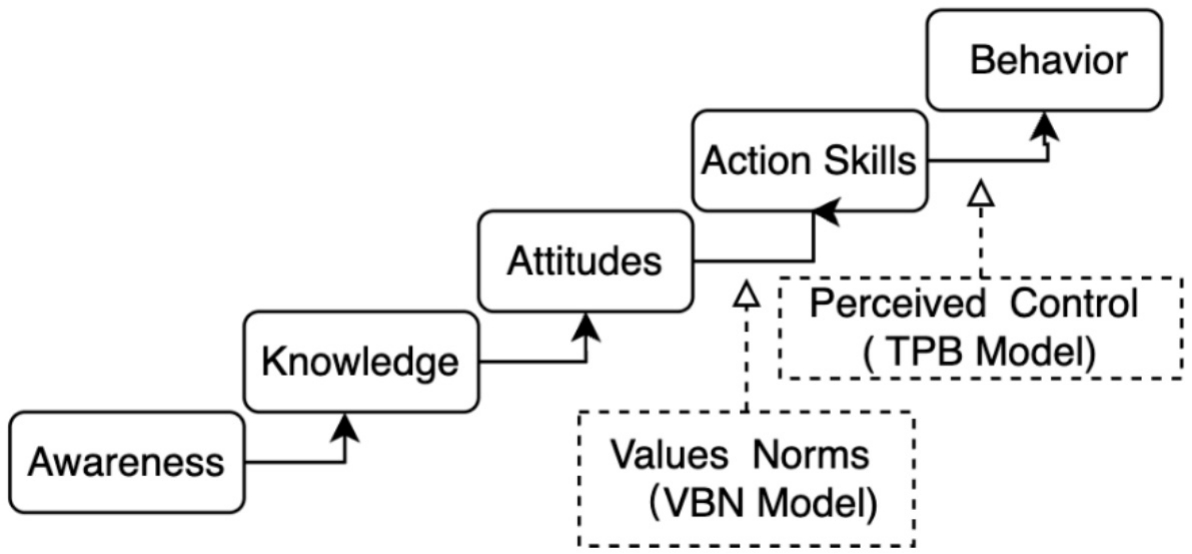
Integrated psychological pathway based on TPB and VBN models. This model illustrates the sequential relationship from awareness to behavior, showing how control beliefs (TPB) and moral norms (VBN) converge to influence pro-environmental action.

In addition to the hypothesized framework, this study also visualizes the underlying psychological mechanisms that integrate the theory of planned behavior (TPB) and the value-belief-norm (VBN) model. As shown in Figure 2, environmental awareness and knowledge sequentially foster attitudes and action skills. Action skills function as a proxy for perceived behavioral control (TPB), while values and norms (VBN) contribute moral motivation that enhances both attitudes and action competencies. This integrative perspective highlights how behavioral intention may be indirectly reflected through action skills, bridging classical theory with practical environmental literacy outcomes.

### 2.3 Integrated research methodology and instrumentation

This study employed a quantitative research design to explore the interrelationships among the five key dimensions of environmental literacy—environmental awareness (EA), environmental knowledge (EK), environmental attitudes (EAT), environmental action skills (EAS), and environmental behavior (EB)—as well as to examine the moderating roles of age, gender, and education level. The conceptual framework and corresponding hypotheses (H1–H9), grounded in the theory of planned behavior (Ajzen, 1991), the value-belief-norm theory (Stern et al., 1999), and the environmental behavior model (Hines et al., 1987), were detailed in Sections 2.1 and 2.2.

A structured questionnaire was developed based on validated environmental literacy surveys previously used in Taiwan, specifically adapted to the context of certified environmental education volunteers. This instrument comprehensively captured all five core dimensions of environmental literacy and collected demographic data, including respondents' age, gender, education level, and occupation. EA, EAT, EAS, and EB were measured using 5-point Likert scales (1 = Strongly Disagree to 5 = Strongly Agree), a widely accepted format for capturing self-reported perceptions, attitudes, and behaviors. EK was assessed through eight objective items, comprising multiple-choice and true/false questions, to evaluate factual understanding of key topics such as air quality, waste management, and ecosystems.

To enhance contextual relevance and engagement, the questionnaire was slightly modified for each region by inserting county-specific references (e.g., “Chiayi County,” “Taichung City,” “Hualien County”) into the item wording while maintaining overall consistency in content and structure. The instrument underwent rigorous expert review by environmental education specialists and social science researchers to ensure clarity, content validity, and alignment with the study's constructs. A pilot test was also conducted with a small group of volunteers to verify readability and make minor adjustments prior to full-scale distribution.

The sampling strategy employed was stratified to ensure balanced representation across age, gender, and education levels. This approach ultimately yielded 921 valid responses (Chiayi: 500; Taichung: 215; Hualien: 206), satisfying Cochran's formula for a 95% confidence level with a ±5% margin of error. Data were analyzed using SPSS (Version 27). Descriptive statistics summarized participant demographics and core study variables. Hierarchical multiple regression was used to examine direct hypotheses (H1–H6), while interaction terms tested the moderating effects (H7–H9). Assumptions of normality, linearity, homoscedasticity, and multicollinearity were thoroughly checked to ensure robustness of analyses.

Hierarchical regression was used to examine the sequential relationships proposed in the environmental literacy model. Variables were entered into the regression in conceptually ordered blocks consistent with the hypothesized pathway: demographic controls (age, gender, and education) were entered first, followed by environmental awareness, knowledge, attitudes, and action skills. This stepwise procedure allowed us to evaluate the incremental variance explained (Δ*R*^2^) and identify the unique contribution of each construct to pro-environmental behavior. All analyses were conducted in SPSS, and standardized coefficients (β), significance levels (*p*-values), and changes in *R*^2^ were reported to assess hypothesis support. Moderation analysis was performed by mean-centering predictor variables, creating interaction terms with demographic variables, and entering these terms into the final regression block. This approach allowed us to examine whether demographic characteristics alter the strength of the relationships among environmental literacy components. Such regression-based techniques are appropriate for testing sequential behavioral pathways and moderation effects in environmental psychology research.

Operational definitions in this study were as follows: EA was measured with eight items addressing key environmental issues; EK with eight factual items; EAT with eight items reflecting beliefs and values; EAS with eight items on practical competencies; and EB with eight items assessing self-reported behaviors. Age, gender, and education served as moderator variables. This rigorous methodological approach provided empirical support to evaluate the framework presented in Figure 1 and laid a strong foundation for cross-regional comparison and hypothesis testing. The complete questionnaire is included in the Appendix.

## 3 Data analysis and key findings

This study purposefully selected three distinct regions in Taiwan—Chiayi, Taichung, and Hualien—to represent diverse socio-geographic contexts and to examine whether environmental literacy pathways are consistent across different community settings. Chiayi, located in southwestern Taiwan, is primarily an agricultural area characterized by rural communities and water resource-based environmental initiatives. Taichung, situated in central Taiwan, is a major urban center with extensive industrial development and a strong focus on air quality and municipal waste management. Hualien, on the eastern coast, presents a semi-urban profile, balancing tourism-driven ecological conservation with agricultural and fishery activities. By including these regions, the study captures a broad spectrum of environmental challenges and volunteer activities, allowing for a robust test of the conceptual framework across varying local conditions. This design enhances the external validity of the findings and provides a nuanced understanding of how regional characteristics may influence the development of environmental literacy among volunteers. This section presents the analysis of the survey data collected from 500 environmental education volunteers in Chiayi, 215 environmental education volunteers in Taichung city, and 206 from Hualien County. The analysis includes descriptive statistics, measurement results, factor analysis and reliability testing, hierarchical regression analysis, and moderation tests. The results provide insights into the relationships between the key dimensions of environmental literacy and the moderating effects of demographic variables such as gender, age, and education level.

### 3.1 Descriptive statistics

Table 1 summarizes the demographic characteristics of environmental education volunteers from Chiayi, Taichung, and Hualien. Gender distribution varied across regions: Chiayi had a balanced sample (54.8% male), while Taichung (62.8%) and Hualien (60.2%) had more female volunteers. Age profiles differed notably: Chiayi's volunteers were older, with 28.8% aged 60–69 and 28.2% aged 50–59. Hualien showed similar trends (30.6% aged 60–69; 17.5% aged 70+), whereas Taichung had a younger profile, with 24.2% aged 20–29 and 17.2% aged 30–39. Across all regions, volunteers under 20 were rare (1%−4%). Educational attainment also varied. In Chiayi, most had completed high school or vocational education (32.4%), while Taichung and Hualien had higher proportions with college or university degrees (51.6 and 50%, respectively). Notably, 34.9% in Taichung held graduate degrees, compared to 5.4% in Chiayi and 9.7% in Hualien. Lower education levels (elementary or junior high) were more common in Chiayi and Hualien. These demographic differences may influence how volunteers perceive, learn about, and act on environmental issues, underlining the importance of considering local contexts when designing educational interventions.

**Table 1:** Volunteers demographic details.

**Category**	**Group**	**Chiayi**		**Taichung**		**Hualien**	
		**Counts**	**% of total**	**Counts**	**% of total**	**Counts**	**% of total**
Gender	Male	274	54.80	80	37.20	82	39.80
	Female	226	45.20	135	62.80	124	60.20
Age	Under 20	5	1.00	8	3.70	37	18.00
	20–29	25	5.00	52	24.20	7	3.40
	30–39	52	10.40	25	11.60	9	4.40
	40–49	71	14.20	27	12.60	20	9.70
	50–59	141	28.20	47	21.90	34	16.50
	60–69	144	28.80	45	20.90	63	30.60
	70 and above	52	10.40	11	5.10	36	17.50
Education level	Uneducated	12	2.40	0	0.00	0	0.00
	Elementary school	85	17.00	1	0.50	11	5.30
	Junior high school	79	15.80	5	2.30	11	5.30
	High school/vocational	162	32.40	23	10.70	61	29.60
	College/University	129	25.80	111	51.60	103	50.00
	Graduate and above	27	5.40	75	34.90	20	9.70
Total	500		215		206		

### 3.2 Measurement results for relevant research variables

Here are the tables for each of the five aspects of environmental literacy, including the questions asked for each aspect in the survey, along with their mean scores and standard deviations:

#### 3.2.1 Environmental awareness (EA)

Table 2 summarizes the descriptive statistics for environmental awareness (EA), measured by eight items assessing perceptions of environmental issues among volunteers in Chiayi, Taichung, and Hualien. Overall, respondents across all counties demonstrated high awareness, with mean scores exceeding 3.6 on a 5-point scale. In Chiayi, the highest mean was for EA8, concerning perceptions of increased climate anomalies (*M* = 4.576, SD = 0.747), followed by EA3 and EA4, which related to recycling and waste sorting. The lowest was EA5 (*M* = 3.982, SD = 0.973), suggesting less certainty about improvements in illegal waste dumping. Taichung showed a similar pattern, with EA8 highest (*M* = 4.726, SD = 0.622), reflecting strong climate change awareness, while EA2 (*M* = 3.595, SD = 1.063) and EA5 (*M* = 3.609, SD = 1.092) were comparatively lower, indicating more moderate views on local environmental management. In Hualien, mean scores were consistently high, particularly for EA4, EA3, and EA8 (means above 4.6), underscoring strong recognition of climate and recycling issues. EA5 remained the lowest (*M* = 3.840, SD = 1.002), consistent with patterns in other counties. Standard deviations ranged from 0.6 to 1.1, suggesting moderate variability without extreme dispersion. These results indicate that environmental education volunteers possess substantial awareness of environmental challenges, especially regarding climate change and personal waste practices.

**Table 2:** Descriptive analysis for the environmental awareness variable.

**Question**	**Chiayi**		**Hualien**		**Taichung**	
	**Mean**	**Standard deviation**	**Mean**	**Standard deviation**	**Mean**	**Standard deviation**
EA1	4.284	0.89	4.252	0.98	4.316	0.877
EA2	4.086	0.978	3.995	1.01	3.595	1.063
EA3	4.528	0.75	4.631	0.712	4.479	0.784
EA4	4.5	0.782	4.675	0.614	4.609	0.688
EA5	3.982	0.973	3.84	1.002	3.609	1.092
EA6	4.3	0.876	4.461	0.781	4.433	0.811
EA7	4.308	0.819	4.257	0.871	3.94	0.972
EA8	4.576	0.747	4.621	0.664	4.726	0.622

#### 3.2.2 Environmental knowledge (EK)

Table 3 shows the descriptive analysis for Environmental Knowledge (EK), assessed via eight multiple-choice or true/false items capturing factual understanding of environmental topics across Chiayi, Taichung, and Hualien. The table reports the percentage of correct responses, highlighting areas of strength and knowledge gaps among volunteers. In Chiayi, participants exhibited strong knowledge of eco-label identification (77.6%), patrol team responsibilities (76.2%), and marine debris composition (75.2%), suggesting familiarity with practical community and pollution issues. However, awareness of the Environmental Education Act was notably limited (0.6%), and lower scores were observed for COVID-19 impacts (37.0%) and air pollution (48.0%). In Hualien, air pollution knowledge was highest (82.5%), followed by marine debris (68.4%) and patrol duties (45.6%). Yet, correct rates were generally lower than in Chiayi, with particularly low scores for COVID-19 impacts (29.6%) and eco-label identification (39.8%). Awareness of the Environmental Education Act was somewhat higher (18.9%), though still modest. Taichung volunteers showed strong understanding of marine debris (79.1%) and moderate awareness of air pollution (59.5%). Other topics, such as wastewater disposal (38.1%), eco-labels (51.6%), and the Environmental Education Act (8.8%), reflected more limited knowledge. The COVID-19 item showed a moderate correct rate (46.5%). Overall, these findings indicate that while volunteers are well-informed on local and practical environmental issues, gaps persist in understanding policy frameworks and emerging environmental health topics. This underscores the need for education programs to integrate policy literacy and current global environmental challenges.

**Table 3:** Descriptive analysis for the environmental knowledge variable.

**Question**	**Chiayi (%)**	**Hualien (%)**	**Taichung (%)**
Air pollution knowledge	48.0	82.5	59.5
Wastewater disposal awareness	66.4	35	38.1
Patrol team responsibility	76.2	45.6	54
Marine debris composition	75.2	68.4	79.1
Food waste recycling knowledge	53.6	36.9	47.4
Eco-label identification	77.6	39.8	51.6
COVID-19 environmental impact	37.0	29.6	46.5
Environmental education act awareness	0.6	18.9	8.8

#### 3.2.3 Environmental attitudes (EAT)

Table 4 presents the descriptive statistics for Environmental Attitudes (EAT), measured through eight items evaluating pro-environmental beliefs, values, and concerns among environmental education volunteers across Chiayi, Hualien, and Taichung. Overall, results indicate consistently strong positive attitudes, with mean scores ranging from 4.19 to 4.72 on a 5-point Likert scale.

**Table 4:** Descriptive analysis for the environmental attitudes variable.

**Question**	**Chiayi**		**Hualien**		**Taichung**	
	**Mean**	**Standard deviation**	**Mean**	**Standard deviation**	**Mean**	**Standard deviation**
EAT1	4.34	0.86	4.62	0.63	4.62	0.64
EAT2	4.19	0.92	4.44	0.82	4.27	0.96
EAT3	4.41	0.83	4.5	0.76	4.45	0.75
EAT4	4.49	0.81	4.65	0.69	4.68	0.73
EAT5	4.43	0.77	4.59	0.68	4.66	0.58
EAT6	4.39	0.81	4.62	0.66	4.72	0.54
EAT7	4.53	0.77	4.67	0.65	4.68	0.57
EAT8	4.36	0.81	4.57	0.66	4.55	0.65

In Chiayi, the highest endorsement was for EAT7 (“Environmental pollution affects food safety”; *M* = 4.53, SD = 0.77), followed by EAT4 (“Using reusable tableware helps reduce waste”; *M* = 4.49, SD = 0.81) and EAT5 (“Addressing climate change issues is essential”; *M* = 4.43, SD = 0.77). The relatively lower score for EAT2 (*M* = 4.19, SD = 0.92), on the acceptability of irrigating with treated livestock wastewater, suggests some reservations tied to health or familiarity concerns.

In Hualien, all EAT items received high agreement (means >4.4), with EAT7 (*M* = 4.67, SD = 0.65) and EAT4 (*M* = 4.65, SD = 0.69) again leading, reflecting a strong consensus on food safety and waste minimization. Standard deviations were the lowest among the three counties (ranging from 0.63 to 0.82), indicating tighter agreement.

Taichung responses were similarly high. The highest means appeared for EAT6 (“Everyone has a responsibility to protect the environment”; *M* = 4.72, SD = 0.54), alongside EAT4 and EAT7 (both *M* = 4.68), highlighting strong personal accountability and concern over pollution's health impacts. As elsewhere, EAT2 remained the lowest (*M* = 4.27, SD = 0.96).

Across counties, standard deviations were generally low to moderate (0.54–0.96), pointing to stable attitudes among volunteers. These findings affirm that volunteers maintain robust pro-environmental values, positioning them as key actors in fostering community sustainability practices.

#### 3.2.4 Environmental action skills (EAS)

Table 5 summarizes the descriptive statistics for Environmental Action Skills (EAS), assessed through eight items capturing volunteers' self-reported capacity to apply environmental knowledge and engage in sustainable practices across Chiayi, Hualien, and Taichung. Overall, mean scores ranged from 3.56 to 4.56, reflecting generally strong confidence in action skills among participants. In Chiayi, the highest mean was for EAS6 (“I can differentiate between general waste, recyclables, and food waste”; *M* = 4.26, SD = 0.90), indicating proficiency in basic sustainability practices. Other high scores included EAS2 (“I take protective measures against air pollution”; *M* = 4.19, SD = 0.86) and EAS7 (“I can recognize eco-labels”; *M* = 4.09, SD = 0.97). The lowest means were for EAS4 (“I document environmental pollution incidents”; *M* = 3.56, SD = 1.14) and EAS5 (“I know how to report pollution to authorities”; *M* = 3.87, SD = 1.09), suggesting less familiarity with formal or technical response skills. In Hualien, volunteers exhibited uniformly high confidence, with all means exceeding 4.0. EAS6 again topped the scale (*M* = 4.56, SD = 0.64), followed by EAS2 (*M* = 4.55, SD = 0.70) and EAS3 (“I participate in community environmental activities”; *M* = 4.38, SD = 0.87). Even the lowest, EAS4 (*M* = 4.10, SD = 0.95), indicated relatively robust engagement in documenting environmental issues. In Taichung, the pattern was similar. EAS2 recorded the highest mean (*M* = 4.53, SD = 0.69), followed by EAS6 (*M* = 4.44, SD = 0.75) and EAS7 (*M* = 4.28, SD = 0.81). As elsewhere, EAS4 received the lowest score (*M* = 3.76, SD = 1.10), underscoring a broader trend across counties that points to potential gaps in pollution documentation and reporting skills.

**Table 5:** Descriptive analysis for the environmental action skills variable.

**Question**	**Chiayi**		**Hualien**		**Taichung**	
	**Mean**	**Standard deviation**	**Mean**	**Standard deviation**	**Mean**	**Standard deviation**
EAS1	3.824	0.991	4.272	0.863	4.288	0.821
EAS2	4.190	0.862	4.549	0.702	4.530	0.689
EAS3	3.894	1.006	4.379	0.868	4.330	0.802
EAS4	3.562	1.144	4.097	0.953	3.763	1.096
EAS5	3.866	1.087	4.286	0.861	4.014	1.021
EAS6	4.264	0.899	4.563	0.643	4.442	0.746
EAS7	4.086	0.966	4.369	0.790	4.279	0.812
EAS8	3.946	0.976	4.345	0.773	4.330	0.813

Collectively, these findings indicate that volunteers are well-equipped with everyday environmental action skills—particularly those related to waste sorting, eco-friendly purchasing, and pollution prevention. However, slightly lower confidence in documenting and formally reporting incidents suggests that these more technical competencies could benefit from targeted capacity-building initiatives.

#### 3.2.5 Environmental behavior (EB)

Table 6 presents the descriptive statistics for Environmental Behavior (EB), measured through eight items assessing the extent to which volunteers integrate pro-environmental actions into their daily routines across Chiayi, Hualien, and Taichung. Overall, mean scores were consistently above 3.8, indicating widespread engagement in sustainable practices. In Chiayi, mean scores ranged from 4.06 to 4.50. The highest was for EB4 (“I regularly sort and recycle household waste”; *M* = 4.50, SD = 0.78), highlighting strong adherence to fundamental recycling behaviors. This was followed by EB2 (“I am willing to replace high-emission vehicles with eco-friendly alternatives”; *M* = 4.30, SD = 0.84) and EB6 (“I report illegal dumping”; *M* = 4.15, SD = 0.99), reflecting both personal and civic environmental responsibility. The lowest mean was for EB3 (“I support buying recycled furniture”; *M* = 4.06, SD = 0.92), suggesting practical or economic considerations may temper enthusiasm for circular consumption. In Hualien, behavioral engagement was uniformly high, with means from 4.21 to 4.68. Again, EB4 received the top rating (*M* = 4.68, SD = 0.63), underscoring the strong culture of household waste sorting. EB2 (*M* = 4.49, SD = 0.73) and EB3 (*M* = 4.35, SD = 0.82) also scored highly, indicating substantial willingness to adopt low-emission transport and purchase eco-friendly products. The relatively low standard deviations (0.63–0.91) across items show a high level of consensus among respondents. In Taichung, overall means remained robust, though slightly more varied. EB4 was again the highest (*M* = 4.63, SD = 0.63), followed by EB2 (*M* = 4.40, SD = 0.78) and EB3 (*M* = 4.35, SD = 0.75). The lowest was EB8 (“I practice low-carbon behaviors such as reducing air conditioner use”; *M* = 3.87, SD = 1.01), possibly reflecting lifestyle or climatic barriers to energy reduction. Across all counties, EB4 consistently stood out as the strongest behavior, highlighting the normalization of recycling among environmental education volunteers. Meanwhile, slightly lower engagement with low-carbon living and eco-product adoption points to areas for future emphasis. These patterns demonstrate how volunteers' environmental knowledge, attitudes, and skills effectively translate into tangible pro-environmental behaviors, reinforcing their role as key agents in local sustainability efforts.

**Table 6:** Descriptive analysis for the environmental behavior variable.

**Question**	**Chiayi**		**Hualien**		**Taichung**	
	**Mean**	**Standard deviation**	**Mean**	**Standard deviation**	**Mean**	**Standard deviation**
EB1	4.15	0.85	4.33	0.81	4.33	0.74
EB2	4.3	0.84	4.49	0.73	4.4	0.78
EB3	4.06	0.92	4.35	0.82	4.35	0.75
EB4	4.5	0.78	4.68	0.63	4.63	0.63
EB5	4.2	0.94	4.35	0.79	4.01	0.97
EB6	4.15	0.99	4.21	0.87	3.77	1.14
EB7	4.13	0.98	4.3	0.78	4.2	0.84
EB8	4.1	0.97	4.22	0.91	3.87	1.01

This study's integrated descriptive analysis of environmental awareness (EA), environmental knowledge (EK), environmental attitudes (EAT), environmental action skills (EAS), and environmental behavior (EB) offers a comprehensive portrait of environmental education volunteers across Chiayi, Hualien, and Taichung. The results collectively reveal a robust environmental literacy profile, albeit with nuanced areas requiring targeted support.

Findings for EA indicate uniformly high awareness of climate change, waste sorting, and recycling, with EA8 (“perceived increase in climate anomalies”) consistently rated highest across counties. Conversely, EA5 (“perceived reduction of illegal dumping”) scored lower, suggesting less confidence in visible outcomes of enforcement efforts.

EK results point to solid knowledge on practical topics—such as eco-labels, marine debris, and local patrol responsibilities—while revealing notable gaps in awareness of formal environmental legislation and emerging issues like COVID-19's environmental impacts. This highlights a need for enhanced dissemination of policy and contemporary environmental information.

Analysis of EAT demonstrates strikingly high mean scores and low variability, underscoring a shared value base emphasizing climate action (EAT5), responsible consumption (EAT4), and health-related environmental concerns (EAT7). Slightly lower agreement on EAT2 (treated wastewater reuse) reflects cautious attitudes toward less familiar sustainability practices.

EAS findings confirm volunteers' confidence in daily pro-environmental tasks, particularly waste sorting (EAS6), air pollution precautions (EAS2), and eco-label recognition (EAS7). However, skills related to documenting and reporting environmental infractions (EAS4, EAS5) consistently lagged, indicating a need to bolster formal action competencies.

EB outcomes show these attitudes and skills effectively translate into behaviors, with highest engagement in recycling (EB4), adopting eco-friendly transportation (EB2), and community reporting (EB6). Lower but still substantial means on purchasing recycled products (EB3) and practicing low-carbon lifestyles (EB8) suggest further encouragement is warranted.

Together, these patterns illustrate that Taiwan's environmental education volunteers possess strong foundational literacy, characterized by acute awareness, shared pro-environmental values, and well-developed personal action skills. Nonetheless, enhancing their formal policy knowledge and capacities for civic or institutional engagement could further empower them as pivotal local sustainability actors. These insights support refining educational initiatives to more strategically address observed gaps, thereby strengthening the long-term efficacy and reach of volunteer-driven environmental programs.

### 3.3 Hierarchical regression analysis

Table 7 reports the hierarchical regression analyses for the six proposed hypotheses (H1–H6), tested separately across Chiayi, Taichung, and Hualien. Overall, all hypothesized relationships were statistically significant (*p* < 0.05), providing strong support for the conceptual framework linking environmental awareness, knowledge, attitudes, action skills, and behavior among environmental education volunteers.

**Table 7:** Hypothesis testing results for hierarchical regression analysis.

**Hypothesis**	**Construct relationship**	**County**	**β coeff**.	***R* ^2^**	***p*-value**	**Result**
H1	Environmental awareness → Environmental knowledge	Chiayi	0.041	0.028	0.000	Supported
		Taichung	0.063	0.012	0.019	Supported
		Hualien	0.097	0.009	0.042	Supported
H2	Environmental knowledge → Environmental attitudes	Chiayi	0.58	0.02	0.002	Supported
		Taichung	0.559	0.274	0.000	Supported
		Hualien	0.59	0.348	0.000	Supported
H3	Environmental knowledge → Environmental action skills	Chiayi	0.665	0.017	0.003	Supported
		Taichung	0.53	0.217	0.000	Supported
		Hualien	0.506	0.256	0.000	Supported
H4	Environmental attitudes → Environmental action skills	Chiayi	0.86	0.491	0.000	Supported
		Taichung	0.659	0.434	0.000	Supported
		Hualien	0.672	0.452	0.000	Supported
H5	Environmental action skills → Environmental behavior	Chiayi	0.771	0.653	0.000	Supported
		Taichung	0.791	0.626	0.000	Supported
		Hualien	0.805	0.648	0.000	Supported
H6	Environmental attitudes → Environmental behavior	Chiayi	0.741	0.617	0.000	Supported
		Taichung	0.764	0.583	0.000	Supported
		Hualien	0.783	0.613	0.000	Supported

In Chiayi, the regression results demonstrated a clear sequential pathway: although the influence of EA on EK (H1) was statistically significant (β = 0.041, *R*^2^ = 0.028), it was relatively modest. However, EK showed notable predictive strength on both EAT (β = 0.580, *R*^2^ = 0.020) and EAS (β = 0.665, *R*^2^ = 0.017). Particularly striking was the high impact of EAT on EAS (β = 0.860, *R*^2^ = 0.491), underscoring that pro-environmental values in Chiayi robustly translated into practical skills. This strong attitudinal effect carried through to behavior as well, with EAT predicting EB (β = 0.741, *R*^2^ = 0.617), and EAS emerging as the most powerful predictor of EB (β = 0.771, *R*^2^ = 0.653).

Taichung revealed a somewhat different pattern. While the path from EA to EK remained significant (β = 0.063, *R*^2^ = 0.012), EK exhibited a particularly strong influence on EAT (β = 0.559, *R*^2^ = 0.274) and a solid impact on EAS (β = 0.530, *R*^2^ = 0.217), indicating that in Taichung, factual environmental knowledge was more deeply linked to shaping both attitudes and skills than in Chiayi. The predictive strength of EAT on EAS (β = 0.659, *R*^2^ = 0.434) was slightly lower than Chiayi, yet still substantial. Meanwhile, EAS was a critical determinant of EB (β = 0.791, *R*^2^ = 0.626), and EAT also directly predicted EB (β = 0.764, *R*^2^ = 0.583), highlighting that both competencies and attitudes significantly drive behavioral outcomes in Taichung.

In Hualien, the relationships showcased both the highest knowledge-to-attitude impact (H2: β = 0.590, *R*^2^ = 0.348) and a very strong link from EK to EAS (β = 0.506, *R*^2^ = 0.256). The influence of EA on EK was also most pronounced here (β = 0.097, *R*^2^ = 0.009) compared to the other counties, suggesting that awareness plays a slightly stronger foundational role. EAT robustly predicted EAS (β = 0.672, *R*^2^ = 0.452), and the path from EAS to EB was exceptionally strong (β = 0.805, *R*^2^ = 0.648), emphasizing that in Hualien, practical environmental competencies are especially pivotal in shaping actual behaviors. EAT similarly had a pronounced effect on EB (β = 0.783, *R*^2^ = 0.613).

When comparing the three counties, Chiayi exhibited the strongest attitudinal influence on skills (H4) and a highly balanced dual path from attitudes and skills to behavior (H6, H5). Taichung showed the most substantial impact of environmental knowledge on shaping both attitudes and skills (H2, H3), reflecting perhaps the younger or more formally educated volunteer base noted in its demographic profile. Hualien consistently displayed the highest predictive strength in translating skills into behavior (H5) and knowledge into attitudes (H2), indicating that both cognitive understanding and skill-building were especially crucial pathways to fostering environmental action in this region.

Together, these findings not only confirm the sequential structure from awareness through to behavior but also highlight subtle regional nuances. They suggest that tailored interventions—whether focused more on strengthening attitudes, deepening knowledge, or building practical skills—may be needed to maximize volunteer impact in different local contexts. This nuanced understanding supports the adaptability and practical application of the environmental literacy framework across diverse community environments.

### 3.4 Moderation test

Moderation analysis was performed to examine whether demographic variables—education level, gender, and age—significantly altered key relationships within the environmental literacy framework. Table 8 summarizes the results of these analyses for the three counties.

**Table 8:** Hypothesis testing results for the moderation hypothesis assessment.

**Hypothesis**	**Construct relationship**	**County**	**β coeff**.	***R* ^2^**	***p*-value**	**Result**
H7	Education × Knowledge → Attitudes	Chiayi	−0.2599	0.0031	0.2111	Not supported
		Taichung	0.8446	0.0036	0.3784	Not supported
		Hualien	−0.0678	0.0002	0.8375	Not supported
H8	Gender × Attitudes → Action Skills	Chiayi	−0.0054	0.0002	0.7475	Not supported
		Taichung	−0.021	0.0056	0.2735	Not supported
		Hualien	0.0194	0.0046	0.3306	Not supported
H9	Age × Awareness → Knowledge	Chiayi	0.0017	0.0002	0.7551	Not supported
		Taichung	0.013	0.0117	0.1146	Not supported
		Hualien	0.0125	0.0043	0.3517	Not supported

Education × Knowledge → Attitudes (H7)

The interaction term assessing whether education moderates the link between environmental knowledge (EK) and attitudes (EAT) was not statistically significant in Chiayi (β = −0.2599, *p* = 0.2111), Taichung (β = 0.8446, *p* = 0.3784), or Hualien (β = −0.0678, *p* = 0.8375). Although the beta directions differed across counties, none reached significance. This suggests that regardless of educational background, volunteers with higher environmental knowledge tend to exhibit similar pro-environmental attitudes. It is possible that shared motivations and similar exposure to environmental initiatives among volunteers reduce the influence of formal education levels.

Gender × Attitudes → Action Skills (H8)

Similarly, gender did not moderate the relationship between attitudes and action skills. The interaction effects were small and non-significant across all counties—Chiayi (β = −0.0054, *p* = 0.7475), Taichung (β = −0.0210, *p* = 0.2735), and Hualien (β = 0.0194, *p* = 0.3306). This indicates that positive environmental attitudes are equally likely to translate into action skills among both male and female volunteers, reflecting a gender-neutral pattern possibly reinforced by collective volunteer norms.

Age × Awareness → Knowledge (H9)

Finally, age did not significantly moderate the relationship between environmental awareness (EA) and knowledge (EK) in any region—Chiayi (β = 0.0017, *p* = 0.7551), Taichung (β = 0.0130, *p* = 0.1146), or Hualien (β = 0.0125, *p* = 0.3517). This suggests that individuals across age groups similarly convert awareness into factual knowledge, likely benefiting from standardized training and community-based learning.

Collectively, these results show that education, gender, and age do not significantly influence the structural pathways linking awareness, knowledge, attitudes, and skills within this volunteer population. The consistency across Chiayi, Taichung, and Hualien points to a stable environmental literacy process unaffected by these demographic differences. This resilience highlights the potential for broadly applicable environmental programs, where shared volunteer experiences and values appear to mitigate demographic disparities—an encouraging outcome for designers of community-based environmental education.

## 4 Discussion

This study examined how five key dimensions of environmental literacy—awareness, knowledge, attitudes, action skills, and behavior—interrelate among environmental education volunteers in Chiayi, Taichung, and Hualien. It also explored whether age, gender, and education moderated these relationships. Descriptive results showed consistently high environmental literacy, especially in attitudes and behavior, though slightly lower scores appeared in technical knowledge and certain action skills, such as reporting violations. This suggests that while volunteers are strongly motivated and value sustainability, there is room to enhance applied competencies and policy understanding.

Hierarchical regression analyses confirmed all six direct hypotheses (H1–H6), supporting the sequential model where awareness leads to knowledge, shaping attitudes and skills, ultimately driving behavior. Action skills emerged as the strongest predictor of behavior, underscoring the importance of practical competencies. In contrast, moderation tests (H7–H9) showed no significant effects of education, gender, or age on these pathways. This implies that within this engaged volunteer population, demographic factors have limited influence on how literacy dimensions connect to produce pro-environmental actions.

### 4.1 Interpretation of direct effects

The analysis of direct pathways among the five core constructs of environmental literacy provides strong empirical support for both the conceptual framework of this study and prior theoretical models. All six hypotheses (H1–H6) were statistically supported across Chiayi, Taichung, and Hualien, reinforcing the notion that environmental literacy is a sequential and interconnected process. Hypothesis 1 (H1) confirmed that Environmental Awareness significantly predicts Environmental Knowledge in all three counties, aligning with the proposition that individuals who are more attuned to environmental problems are also more inclined to pursue factual understanding and informational depth. This relationship underscores longstanding perspectives on awareness as a critical motivational antecedent to knowledge acquisition (Tikka et al., 2000). Hypothesis 2 (H2) established that Environmental Knowledge positively influences Environmental Attitudes. This finding resonates with the theory of planned behavior (Ajzen, 1991), which posits that informed cognitive assessments shape attitudinal orientations. It is further corroborated by empirical research indicating that an expanded knowledge base reinforces individuals' evaluative frameworks toward sustainability (Varela-Candamio et al., 2018). Hypotheses 3 (H3) and 4 (H4) highlighted the roles of knowledge and attitudes in shaping Environmental Action Skills. Both pathways were significant, illustrating that a solid cognitive foundation (knowledge) coupled with strong pro-environmental values (attitudes) translates into higher confidence and capability to engage in practical environmental actions. This pattern is consistent with models suggesting that procedural competence arises from the interplay of informational clarity and motivational commitment (Kaiser and Fuhrer, 2003). Hypothesis 5 (H5) demonstrated the strongest effect across counties, showing that Environmental Action Skills robustly predict Environmental Behavior. This outcome validates the critical notion that behavior change in the environmental domain is not solely intention-driven but heavily reliant on individual capacity and skill mastery. It echoes prior findings that well-developed action skills significantly enhance the likelihood of consistent pro-environmental practices (Frick et al., 2004). The strong predictive effect of action skills in this study can be understood through their close relationship with self-efficacy and perceived behavioral control. Action skills represent the procedural capabilities that enable volunteers to translate environmental intentions into concrete tasks such as conducting ecological patrols, documenting environmental changes, or leading community activities. When individuals possess these practical competencies, they also experience a higher sense of confidence and control over performing the behavior—two key components of self-efficacy. This enhanced confidence reduces perceived barriers and increases the likelihood of behavioral execution, which aligns with the theory of planned behavior's emphasis on perceived behavioral control as a precursor to action. Prior research also indicates that individuals with higher procedural competence are more capable of sustaining environmental behaviors because they feel more capable of influencing outcomes. Thus, action skills do not simply reflect technical abilities; they operate as psychological enablers that strengthen one's belief in their ability to act, thereby explaining why they emerged as the strongest predictor of pro-environmental behavior in the volunteer context. Finally, Hypothesis 6 (H6) confirmed that Environmental Attitudes significantly predict Environmental Behavior, affirming the enduring view that affective and normative commitments are foundational precursors to action, especially when infrastructural and knowledge barriers are minimized (Kollmuss and Agyeman, 2002). Collectively, these results reinforce the sequential progression embedded in many environmental literacy frameworks—from awareness leading to knowledge, from knowledge to attitudes and skills, and ultimately culminating in observable behavior. The consistency of these findings across distinct regional contexts in Taiwan underscores the robustness and applicability of this conceptual pathway in guiding environmental education interventions.

### 4.2 Moderation analysis discussion

The moderation analysis examined whether education level, gender, and age influenced the strength of key pathways within the environmental literacy model. Although prior studies have frequently reported demographic variations in environmental engagement (Milfont and Duckitt, 2010; Vicente-Molina et al., 2013), none of the moderation hypotheses (H7–H9) were statistically supported in Chiayi, Taichung, or Hualien. For Hypothesis 7 (H7), education level did not moderate the relationship between Environmental Knowledge and Attitudes. This suggests that regardless of formal educational attainment, volunteers with greater environmental knowledge are equally likely to develop positive environmental attitudes. While this contrasts with findings from general population studies, it may reflect the homogenizing influence of volunteer settings, where participants share similar training experiences and environmental commitments. Hypothesis 8 (H8) tested whether gender moderated the pathway from Environmental Attitudes to Action Skills. Results showed no significant gender-based differences in how attitudes translated into practical environmental competencies across any of the three counties. This implies that both male and female volunteers similarly convert their pro-environmental attitudes into actionable skills, potentially due to the inclusive and egalitarian nature of volunteer programs, which can minimize traditional gender disparities. Hypothesis 9 (H9) evaluated whether age moderated the relationship between Environmental Awareness and Knowledge. Again, no significant moderation was found, indicating that environmental awareness fosters knowledge acquisition consistently across age groups. This outcome may stem from the experiential and participatory learning approaches commonly employed in volunteer contexts, which help equalize learning outcomes regardless of age (Ballantyne and Packer, 2005). Collectively, these findings indicate that demographic characteristics such as education, gender, and age exert minimal influence on the structural pathways of environmental literacy within volunteer populations. This highlights the standardizing effect of volunteer engagement, where shared motivations, collaborative experiences, and structured environmental training likely reduce the impact of individual demographic differences.

### 4.3 Practical and policy implications

This study offers several valuable insights for the design and enhancement of environmental education programs, particularly those tailored for volunteer groups. The validated direct pathways among dimensions of environmental literacy underscore the importance of an integrated, sequential educational model. Programs should purposefully guide participants from raising awareness, to building knowledge, reinforcing pro-environmental attitudes, cultivating practical action skills, and ultimately fostering sustained behavioral change. Notably, the critical bridging role of Environmental Action Skills highlights the need for hands-on learning components. Educational initiatives should incorporate practical exercises such as role-plays, community simulations, and field-based activities that bolster confidence in essential competencies, including recycling, pollution surveillance, and formal environmental reporting. Such experiential approaches help close the well-documented intention–behavior gap, translating cognitive and affective readiness into consistent pro-environmental practices (Frick et al., 2004; Kollmuss and Agyeman, 2002). The lack of significant moderation by demographic factors implies that well-structured environmental education programs can be effectively implemented across diverse participant profiles without necessitating extensive customization. This supports the scalability of volunteer-centered approaches and suggests that inclusive, community-based learning environments may inherently reduce traditional divides linked to education, gender, or age. Nevertheless, maintaining consistent program quality, equitable access to learning resources, and fair opportunities for participation remains essential to uphold these benefits. Additionally, the robust levels of pro-environmental attitudes observed among volunteers point to a substantial intrinsic motivation toward sustainability. Policymakers and non-governmental organizations should leverage this commitment by systematically embedding volunteers into local environmental governance processes. Volunteers could act as community monitors, peer educators, or grassroots policy advocates, thereby extending the reach and amplifying the impact of broader environmental initiatives. Finally, these findings carry implications for national environmental literacy benchmarking. By systematically assessing each component of environmental literacy across key societal groups, Taiwan can design more targeted strategies to advance environmental outcomes and more effectively contribute to global objectives related to sustainability, climate adaptation, and carbon neutrality.

### 4.4 Theoretical implications for TPB and VBN integration

This study contributes to the advancement of behavioral theories by integrating the TPB and VBN framework, with empirical evidence drawn from hierarchical regression analyses across three counties in Taiwan. The quantitative results not only validate the hypothesized relationships but also provide nuanced insights into the underlying mechanisms of pro-environmental behavior among environmental education volunteers.

From a TPB perspective, environmental action skills (EAS) emerged as a pivotal construct that bridges attitudes and behavior. Across all three counties, the regression coefficients linking EAS to pro-environmental behavior were consistently high (β = 0.771 in Chiayi, β = 0.791 in Taichung, and β = 0.805 in Hualien; all *p* < 0.001), with *R*^2^ values exceeding 0.62. These findings suggest that perceived behavioral control—traditionally conceptualized as self-efficacy or perceived ease of performing a behavior—may, in volunteer contexts, be better represented through embodied action skills. Volunteers with high procedural competence may not require explicit behavioral intention to act sustainably; rather, their well-developed skills inherently enable them to translate attitudes into actions.

In addition, the strong predictive path from environmental attitudes to environmental behavior (H6) in all counties (β ranging from 0.741 to 0.783, *p* < 0.001) aligns with the norm activation component of the VBN theory. This affirms that moral and affective drivers—such as personal norms and ethical concern for the environment—remain central to behavior formation, particularly when technical or structural barriers are minimized within volunteer communities.

The results also support a sequential integration of TPB and VBN components. Environmental knowledge (EK), driven by awareness (H1), significantly predicts both attitudes (H2) and action skills (H3), while attitudes (H4, H6) influence both skills and behavior. This layered pathway reveals that knowledge and skills function as cognitive and procedural enablers, while attitudes and norms act as motivational levers—reflecting the dual-process structure of sustainable action.

Importantly, the absence of moderation effects by gender, age, or education (H7–H9) suggests that the TPB and VBN pathways operate uniformly across demographic groups in structured volunteer settings. This reinforces the hypothesis that communal learning environments can buffer individual variability, fostering consistent psychological mechanisms regardless of background.

Overall, this study refines both theoretical models by demonstrating that environmental action skills are not merely derivative outcomes, but rather active drivers in the behavioral pathway. This points to a need for greater emphasis on skills-based design in educational interventions and encourages theoretical frameworks to incorporate procedural control more explicitly alongside cognitive and affective constructs.

### 4.5 Limitations and future research directions

Despite the empirical robustness of this study, several limitations warrant consideration. First, the sample was limited to environmental education volunteers across three counties in Taiwan. While this group offers valuable insights into pro-environmental behavior within structured, motivated communities, the findings may not generalize to the broader public or to populations with lower baseline environmental engagement. Future research should therefore include more heterogeneous samples, including students, general residents, or policy stakeholders, to assess the external validity of the proposed model.

Second, the cross-sectional design restricts causal interpretation. Although the hypothesized pathways are grounded in established theories such as the theory of planned behavior (TPB) and the value-belief-norm (VBN) model, longitudinal studies or intervention-based experiments are necessary to determine the temporal sequencing and durability of environmental literacy development over time.

Third, while this study tested key demographic moderators (education, gender, and age), other potential influences were not examined. Factors such as volunteer tenure, environmental identity, and personal value orientations may shape the strength or salience of the literacy-behavior pathways. Incorporating such psychosocial variables would enrich understanding of the differential mechanisms that drive pro-environmental action.

Fourth, the integration of action skills into TPB and VBN frameworks represents a conceptual innovation, yet further validation is needed. While this study found action skills to be the strongest predictor of behavior (β = 0.77–0.81 across counties), future research should explore how these skills develop, how they interact with perceived behavioral control, and whether they serve similar bridging roles in other behavioral domains (e.g., energy conservation, biodiversity support).

In addition, although hierarchical regression was appropriate for examining the sequential pathways and moderation effects in this study, future research could employ Partial Least Squares Structural Equation Modeling (PLS-SEM) to test latent constructs and evaluate the integrated TPB–VBN framework within a full structural model. This would allow researchers to validate measurement models and assess theoretical relationships simultaneously, offering deeper insight into the robustness of the proposed pathways.

Finally, cultural, and contextual factors may moderate the efficacy of the integrated TPB–VBN model. Taiwan's civic volunteer structure, environmental policy emphasis, and regional environmental programs may all shape behavior differently than in other socio-political contexts. Comparative cross-cultural studies could determine whether the model holds across different governance systems, educational norms, and environmental challenges.

Collectively, these limitations offer pathways for refinement, replication, and theoretical advancement. Future research that expands population scope, employs longitudinal design, and integrates deeper psychosocial variables will further elucidate how pro-environmental behavior can be reliably cultivated through education and community engagement.

Methodologically, future research could also adopt multi-method modeling strategies to capture potentially non-linear and configurational relationships among psychological constructs. For instance Cai et al. (2025b) combined PLS-SEM, artificial neural networks (ANN), and fuzzy-set qualitative comparative analysis (fsQCA) to analyze university students' flexible employment intentions, showing how linear effects, variable importance, and multiple causal pathways can be examined within a single research design. Similarly, Cai et al. (2025a) applied a PLS-SEM–ANN framework to deepen understanding of self-determined efficacy and environmental conservation intentions. Although the present study relies on hierarchical regression, our findings provide a theoretically grounded baseline that subsequent studies can advance using such multi-method approaches to more fully explore complex interactions among environmental literacy, norms, and behavioral outcomes.

Additionally future research should employ longitudinal or experimental approaches to track how environmental literacy evolves and explore additional factors—such as intrinsic motivations or engagement duration—that might influence the pathway from awareness to sustained action. Including broader community stakeholders would also deepen understanding of how diverse groups contribute to, and benefit from, environmental literacy.

## 5 Conclusion

This study explored how five core dimensions of environmental literacy—awareness, knowledge, attitudes, action skills, and behavior—interrelate among environmental education volunteers in Taiwan, using the theory of planned behavior (TPB) and value-belief-norm (VBN) frameworks. Data from 921 volunteers across three counties confirmed a sequential model: awareness fosters knowledge, shaping attitudes and skills, which ultimately drive pro-environmental behavior. Action skills stood out as the strongest predictor, emphasizing the critical role of practical competencies in bridging the intention–behavior gap.

The findings extend classical behavioral models by showing that TPB and VBN pathways operate robustly in volunteer-based education across varied regional and demographic profiles. The absence of significant moderation by education, gender, or age suggests that structured volunteer environments may help equalize differences often seen in broader populations, underscoring the power of community-based experiential learning. Practically, these results highlight the need for programs that combine knowledge-building with hands-on training in skills like monitoring and community engagement. The consistency of outcomes across urban and rural areas supports the scalability of such interventions. Furthermore, leveraging volunteers' strong environmental values can accelerate skill development and behavioral change, amplifying local sustainability efforts. In sum, this study demonstrates that integrated, skill-focused environmental education can cultivate robust literacy frameworks that transcend demographic divides, offering a valuable foundation for designing inclusive strategies to advance local and global sustainability goals.

## Data Availability

The raw data supporting the conclusions of this article will be made available by the authors, without undue reservation.
