# Supplementary material for: Psychological determinants of pro-environmental behavior among environmental volunteers in Taiwan: a TPB and VBN approach
Source: Front Psychol. 2026 Jan 21;16:1670383. doi: 10.3389/fpsyg.2025.1670383 (PMC12867903; doi:10.3389/fpsyg.2025.1670383)
Supplement: Supplementary file 1 [file Data_Sheet_1.docx]

**Questionnaire Survey**

**English version:**

- **Basic Information**

Please check (✔) in the appropriate box and fill in the required information for each of the following questions.

1. **Gender:** 🞏 Male 🞏 Female
2. **Age**: 🞏 Under 20 years old 🞏 20-29 years old 🞏 30-39 years old 🞏 40-49 years old 🞏 50-59 years old 🞏 60-69 years old 🞏 70 and above 🞏 Refuse to answer
3. **Profession**: 🞏 Manufacturing 🞏 Education 🞏 Service industry 🞏 Military police, civil servants 🞏 Financial and insurance industries 🞏 Agriculture, forestry, fishery and animal husbandry □Electricity and gas supply industry 🞏Water supply and pollution control industry 🞏 Retired/housekeeper/unemployed 🞏 Others (please fill in the description) _____
4. **Education**: 🞏 Uneducated 🞏Elementary school 🞏 Junior high school□Senior high school 🞏 College 🞏 Graduate school or above

- **Questionnaire**

Please indicate your agreement with the following question using the following scale: 1 for strongly disagree, 2 for disagree, 3 for neutral, 4 for agree, and 5 for strongly agree.

| 1. **Environmental Awareness (EA)** | 1 | 2 | 3 | 4 | 5 |
| --- | --- | --- | --- | --- | --- |
| 1. I can recognize environmental pollution issues in Chiayi County (e.g., air pollution, abnormal river water color, street litter). |  |  |  |  |  |
| 1. I have observed improvements in environmental quality in Chiayi County in recent years (e.g., reduced open-air burning, improved river water quality). |  |  |  |  |  |
| 1. Practicing waste recycling habits contributes to environmental cleanliness and waste reduction. |  |  |  |  |  |
| 1. I can perceive the positive impact of waste sorting (general waste, recyclables, kitchen waste) on the environment. |  |  |  |  |  |
| 1. I have noticed a reduction in illegal waste dumping in Chiayi County. |  |  |  |  |  |
| 1. I can recognize the positive environmental impact of using or purchasing eco-labeled green products. |  |  |  |  |  |
| 1. I have noticed that Chiayi County is hosting more environmental education and awareness events than in the past. |  |  |  |  |  |
| 1. I feel that climate anomalies have become more frequent in recent years. |  |  |  |  |  |

Please check (✔) the box that you believe is the correct answer based on the issue described in the following text.

| 1. **Environmental Knowledge (EK)** |
| --- |
| 1. PM10 has a greater concentration in the air than PM2.5 and poses a more serious health risk. 🞏 True 🞏 False |
| 1. Household wastewater, after treatment at a sewage plant, can be directly discharged into rivers. 🞏 True 🞏 False |
| 1. Which of the following is not a responsibility of the Chiayi County Water Environment Patrol Team?   🞏 Water quality monitoring activities 🞏 Aquatic ecological surveys 🞏 Environmental education and advocacy  🞏 Assisting with soil and water conservation engineering |
| 1. In recent years, which of the following categories of marine debris accounted for the highest proportion in the results of underwater garbage removal in Chiayi County?   🞏 Fishing and recreational fishing 🞏 Smoking-related waste 🞏 Medical/personal hygiene products 🞏 Household waste and recreational activities |
| 1. Which of the following is not a benefit of food waste recycling? Which of the following is not a benefit of food waste recycling? 🞏 Prevents household garbage from rotting and breeding mosquitoes and flies, improving environmental hygiene. 🞏 Reduces the burden of waste treatment and lowers expenses for incineration plants and landfills. 🞏 Can be converted into animal feed to solve food supply issues for sheltered animals. 🞏 Cultivates good habits among citizens to cherish resources |
| 1. Which of the following label and name pairings is incorrect? 🞏 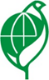Environmental Protection Label (環保標章) 🞏 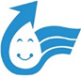Water-Saving Label (省水標章) 🞏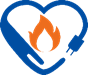 Energy-Saving Label (節能標章) 🞏 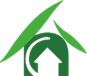Green Building Label (綠建築標章) |
| 1. What is the primary environmental pollution caused by the COVID-19 pandemic? Which of the following is a major type of environmental pollution that accompanied the COVID-19 pandemic? 🞏Air pollution 🞏Garbage pollution 🞏 Noise pollution 🞏Water pollution |
| 1. The Environmental Education Act was passed in 2010. Which of the following does not align with its promotion principles? 🞏 Maintaining ecological balance, respecting life, and promoting social justice. 🞏Enhancing human well-being to achieve sustainable development. 🞏Understanding the interdependent relationship between human society and nature. 🞏Enhancing public awareness, environmental ethics, and responsibility. |

Please indicate your agreement with the following question using the following scale: 1 for strongly disagree, 2 for disagree, 3 for neutral, 4 for agree, and 5 for strongly agree.

| 1. **Environmental Attitudes (EAT)** | 1 | 2 | 3 | 4 | 5 |
| --- | --- | --- | --- | --- | --- |
| 1. I prioritize environmental protection more than before. |  |  |  |  |  |
| 1. I support the use of treated livestock wastewater for crop irrigation. |  |  |  |  |  |
| 1. More river water monitoring devices should be installed to prevent pollution. |  |  |  |  |  |
| 1. Using reusable tableware helps reduce waste. |  |  |  |  |  |
| 1. Addressing climate change issues is essential. |  |  |  |  |  |
| 1. Purchasing local and seasonal agricultural products reduces energy loss. |  |  |  |  |  |
| 1. Environmental pollution affects food safety. |  |  |  |  |  |
| 1. I prefer purchasing eco-friendly products that emphasize sustainability. |  |  |  |  |  |

Please indicate your agreement with the following question using the following scale: 1 for strongly disagree, 2 for disagree, 3 for neutral, 4 for agree, and 5 for strongly agree.

| 1. **Environmental Action Skills (EAS)** | 1 | 2 | 3 | 4 | 5 |
| --- | --- | --- | --- | --- | --- |
| 1. I can interpret air quality index colors (e.g., hazardous levels). |  |  |  |  |  |
| 1. I take protective measures against air pollution. |  |  |  |  |  |
| 1. I encourage colleagues to reduce waste by using eco-friendly materials. |  |  |  |  |  |
| 1. I document environmental pollution incidents (e.g., taking photos, videos). |  |  |  |  |  |
| 1. I know how to report environmental pollution to authorities. |  |  |  |  |  |
| 1. I can differentiate between general waste, recyclables, and food waste. |  |  |  |  |  |
| 1. I can recognize commonly used eco-labels. |  |  |  |  |  |
| 1. I can discuss environmental topics accurately with friends. |  |  |  |  |  |

Please indicate your agreement with the following question using the following scale: 1 for strongly disagree, 2 for disagree, 3 for neutral, 4 for agree, and 5 for strongly agree.

| 1. **Environmental Behavior (EB)** | 1 | 2 | 3 | 4 | 5 |
| --- | --- | --- | --- | --- | --- |
| 1. I actively learn about environmental pollution prevention. |  |  |  |  |  |
| 1. I am willing to replace high-emission vehicles with eco-friendly alternatives. |  |  |  |  |  |
| 1. I support buying recycled furniture. |  |  |  |  |  |
| 1. I regularly sort and recycle household waste. |  |  |  |  |  |
| 1. I participate in community clean-up activities. |  |  |  |  |  |
| 1. I intervene when I see illegal waste dumping. |  |  |  |  |  |
| 1. I purchase eco-friendly products even if they cost more. |  |  |  |  |  |
| 1. I attend environmental awareness events in Chiayi County. |  |  |  |  |  |

**Chinese version:**

- **基本資料**

請您在下列各題適當欄位中打「V」和填寫資料

1. **性別**：🞏男 🞏女
2. **年齡：**🞏 20歲以下　🞏 20-29歲 　🞏 30-39歲 　🞏 40-49歲 🞏 50-59歲 　🞏 60-69歲　🞏 70以上 　🞏拒絕回答
3. **職業：** 🞏 製造業 🞏 教育業 🞏 服務業 🞏軍警、公務人員 🞏 金融及保險業 🞏 農、林、漁、牧業 🞏 電力及燃氣供應業 🞏用水供應及污染整治業 🞏 退休/家管/待業中 🞏其他（請填寫說明）
4. **教育程度**：🞏未受教育 🞏 國小 🞏 國中 🞏 高中高職 🞏大專大學 🞏研究所以上
   - **問卷調查**

請您依據下列量表對以下問題表示同意程度：1 表示非常不同意，2 表示不同意，3 表示普通，4 表示同意，5 表示非常同意。

| 1. **環境覺知與敏感度（Environmental Awareness, EA）** | 1 | 2 | 3 | 4 | 5 |
| --- | --- | --- | --- | --- | --- |
| 1. 我能注意到嘉義縣的環境污染問題。（例如空氣混濁、河川水色異常、街道垃圾等。） |  |  |  |  |  |
| 2. 我發現嘉義縣近年來的環境品質獲得改善。（例如露天燃燒、河川水質優化情形減少等。） |  |  |  |  |  |
| 3. 我發現執行資源回收習慣有助於環境整潔與垃圾減量。 |  |  |  |  |  |
| 4. 我能察覺執行垃圾分類(一般垃圾、資源回收、廚餘回收) 對於環境的正面影響。 |  |  |  |  |  |
| 5. 我發現嘉義縣廢棄物非法棄置情況減少了。 |  |  |  |  |  |
| 6. 我能察覺使用或購買具有環保標章的綠色產品對於環境有正面影響。 |  |  |  |  |  |
| 7. 我發現嘉義縣比起過往舉辦更多包含環境教育及環境保護宣導的活動。 |  |  |  |  |  |
| 8. 我感覺近年來氣候異常情況變得頻繁。 |  |  |  |  |  |

請依照下列文字敘說中所闡述的問題，在□處勾選您認為的正確答案。

| 1. **環境知識（Environmental Knowledge, EK）** |
| --- |
| 9. 空氣中的PM10含量大於PM2.5，其對人體的危害也較為嚴重。🞏 對 🞏錯 |
| 10. 只要經過污水處理廠處理過的家庭廢水，就能直接排放至河流。🞏 對 🞏錯 |
| 11. 下列何者非為嘉義縣水環境巡守隊的工作項目？🞏水質監測活動 🞏水域生態調查 🞏 環境教育宣導 🞏協助水保工程 |
| 12. 近年來嘉義縣海底垃圾清除結果中，占比最高為下列何者垃圾分類？🞏 漁業與休閒釣魚 🞏 抽菸行為 🞏醫療/個人衛生用品 🞏生活垃圾與遊憩行為 |
| 13. 下列何者非廚餘回收的好處？🞏 避免家中垃圾腐敗及孳生蚊蠅，改進環境衛生 🞏 降低廢棄物處理負荷，同時減少焚化廠及掩埋場支出 🞏 可再製成飼料，解決收容動物食物來源 🞏 養成國人珍惜資源之良好習慣 |
| 14. 下列標章與名稱和者配對錯誤？🞏 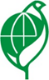環保標章 🞏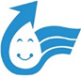省水標章 🞏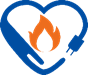 節能標章 🞏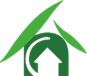 綠建築標章 |
| 15. 下列何者為因新冠肺炎 (COVID-19) 伴隨而來的主要環境污染？🞏空氣污染 🞏垃圾污染 🞏 噪音污染 🞏水質污染 |
| 16. 2010年通過了環境教育法，下列何者不符合推動原則？🞏 維護生態平衡、尊重生命、促進社會正義。🞏增進人類的生活福祉，以達到永續發展。🞏了解人類社會與自然之間相互依存的關係。🞏 增進全民環境認知、環境倫理與責任。 |

請您依據下列量表對以下問題表示同意程度：1 表示非常不同意，2 表示不同意，3 表示普通，4 表示同意，5 表示非常同意。

| 1. **環境態度（Environmental Attitudes, EAT）** | 1 | 2 | 3 | 4 | 5 |
| --- | --- | --- | --- | --- | --- |
| 17. 你認同「我比以前更重視環境保護議題，例如環境污染、棲地破壞、食安等」。 |  |  |  |  |  |
| 18. 你認同「使用經處理過的畜牧廢水於澆灌農作物，以減少化學肥料使用」。 |  |  |  |  |  |
| 19. 你認同「應該多設置河川水質監測設備，監測嘉義縣河川水質變化來避免河川污染」。 |  |  |  |  |  |
| 20. 你認同「使用環保餐具能減少一次性廢棄物(例如：免洗餐具)的產生」。 |  |  |  |  |  |
| 21. 你認同「主動關心氣候變遷所帶來的環境問題是重要的」。 |  |  |  |  |  |
| 22. 你認同「購買當地當季農產品可以減少運輸、保存的能源損耗」。 |  |  |  |  |  |
| 23. 你認同「環境污染會間接或直接的影響到食安問題」。 |  |  |  |  |  |
| 24. 你認同應購買強調環境保護的綠色商品，如嘉有好市集的在地農產品」。 |  |  |  |  |  |

請您依據下列量表對以下問題表示同意程度：1 表示非常不同意，2 表示不同意，3 表示普通，4 表示同意，5 表示非常同意。

| 1. **環境行動技能（Environmental Action Skills, EAS）** | 1 | 2 | 3 | 4 | 5 |
| --- | --- | --- | --- | --- | --- |
| 25. 我能分辨空氣品質指數(AQI)的顏色所代表的意涵與意義，例如紫爆、紅害等。 |  |  |  |  |  |
| 26. 我能針對空氣污染，採取防護方法(例如：戴上口罩、使用空氣清淨設備、減少外出)以保護自身健康。 |  |  |  |  |  |
| 27. 為了垃圾源頭減量，我會邀請同事自備環保餐具及容器去購買飲料、餐食。 |  |  |  |  |  |
| 28. 遇到環境污染問題，我會進行蒐證，例如拍照、錄影、錄音等。 |  |  |  |  |  |
| 29. 遇到環境污染問題，我知道如何向有關單位提出通報。 |  |  |  |  |  |
| 30. 我能清楚的分辨一般垃圾、資源回收與生/熟廚餘。 |  |  |  |  |  |
| 31. 我能識別常見的環保標章。 |  |  |  |  |  |
| 32. 我能傳達正確的環保相關資訊並跟朋友談論環保問題。 |  |  |  |  |  |

請您依據下列量表對以下問題表示同意程度：1 表示非常不同意，2 表示不同意，3 表示普通，4 表示同意，5 表示非常同意。

| 1. **環境行動力（Environmental Behavior, EB）** | 1 | 2 | 3 | 4 | 5 |
| --- | --- | --- | --- | --- | --- |
| 33. 我會主動學習有關環境污染的新知識及防治方法。 |  |  |  |  |  |
| 34. 我願意主動汰換高污染車輛(例如：二行程車、烏賊車)。 |  |  |  |  |  |
| 35. 我會支持購置再生家具。 |  |  |  |  |  |
| 36. 我會主動落實垃圾分類工作。 |  |  |  |  |  |
| 37. 當我發現社區有垃圾時，我會去協助清理。 |  |  |  |  |  |
| 38. 當我發現社區有人偷倒垃圾時，我會上前去勸說。 |  |  |  |  |  |
| 39. 我會購買具有環保標章的產品，即便單價可能較高。 |  |  |  |  |  |
| 40. 我會主動參與嘉義縣所舉辦的各類環保活動。 |  |  |  |  |  |
